# Reproducibility and utility of an overnight 0.25 mg dexamethasone suppression test as a marker for glucocorticoid sensitivity in children with asthma

**DOI:** 10.1007/s40618-015-0323-6

**Published:** 2015-06-10

**Authors:** R. H. Willemsen, L. van Leeuwen, T. A. S. Voorend-van Bergen, Y. B. de Rijke, M. W. Pijnenburg, E. L. T. van den Akker

**Affiliations:** Department of Paediatric Endocrinology, Erasmus MC Sophia, 3015 GJ Rotterdam, The Netherlands; Department of Paediatric Pulmonology, Erasmus MC Sophia, 3015 GJ Rotterdam, The Netherlands; Diagnostic Laboratory Endocrinology, Departments of Clinical Chemistry and Internal Medicine, Erasmus MC, 3015 GJ Rotterdam, The Netherlands; Department of Paediatrics, Box 116 Level 8 Addenbrooke’s Hospital, University of Cambridge, Cambridge Biomedical Campus, Hills Road, Cambridge, CB2 0QQ UK

**Keywords:** Glucocorticoid sensitivity, Paediatric endocrinology, Asthma

## Abstract

**Purpose:**

Inhaled corticosteroids (ICS) are the cornerstone of asthma treatment in children. However, there is considerable inter-individual variation in glucocorticoid sensitivity, leading to over- as well as undertreatment. A simple and fast test to predict glucocorticoid sensitivity would enable more tailored therapy in children with asthma.

**Aim:**

To study reproducibility and utility of an overnight 0.25 mg dexamethasone suppression test (DST) with salivary cortisol levels as marker for glucocorticoid sensitivity in asthmatic children.

**Methods:**

23 children with atopic asthma were recruited for two overnight 0.25 mg DST’s, 1 month apart.

**Results:**

Baseline cortisol levels correlated well between both tests. However, cortisol levels, change in cortisol levels or fractional suppression of cortisol levels after dexamethasone did not correlate between the two tests. Bland–Altman plots showed that the difference in salivary cortisol levels between test 1 and 2 of an individual patient could go up to 12 nmol/l, which is a clinically relevant difference. ICS dose did not correlate with baseline cortisol levels, height and BMI SDS.

**Conclusion:**

The low-dose salivary DST test in its current form is not suitable for use in clinical practice in children with asthma, due to low reproducibility. Therefore, studies using the 0.25 mg salivary DST should be interpreted cautiously.

## Introduction

Inhaled corticosteroids (ICS) are the cornerstone of treatment in children with persistent asthma [1]. However, there is considerable inter-individual variation regarding the response to ICS and side effects. Overtreatment with ICS may decrease height velocity, increase weight and induce (partial) adrenal insufficiency, whereas undertreatment may cause persisting asthmatic complaints, exacerbations, unscheduled health care visits and reduced quality of life. A simple and fast test to predict glucocorticoid sensitivity would enable more tailored therapy.

The 0.25 mg dexamethasone suppression test (DST) was developed to assess individual glucocorticoid sensitivity [2]. As opposed to the 1 mg DST (used to diagnose Cushing’s syndrome), the 0.25 mg DST only partially suppresses cortisol levels. The Gaussian distribution of post-dexamethasone cortisol levels in 164 healthy adults reflects inter-individual differences in glucocorticoid sensitivity [2]. Hypersensitive persons suppress their HPA-axis more in the 0.25 mg DST than resistant persons.

Our objectives were (1) to investigate the reproducibility of an overnight 0.25 mg DST, using salivary cortisol levels, in children with asthma and (2) to investigate the utility of this test as a marker for glucocorticoid sensitivity.

## Methods

This pilot study was embedded in the multi-centre study ‘Better Asthma Treatment: Monitoring with Asthma Control Test (ACT) and Nitric oxide’ (BATMAN) [3]. The local Ethics Committee approved the study. All patients (when aged ≥12 years) and parents gave informed consent.

23 Children with atopic asthma sampled saliva in the morning, ingested dexamethasone in the evening and provided a second sample the morning thereafter. To evaluate reproducibility, the same test was repeated after 1 month in 16 children, without changing the ICS dose.

Participants discontinued ICS and topical corticosteroids, during test days only. The dexamethasone dose (based on the 0.25 mg DST for adults) was adjusted to the children’s weight (3.33 µg/kg bodyweight, rounded off to 50 µg units; maximum dose 0.25 mg).

Salivary cortisol levels were measured using a commercial enzyme immunoassay (DRG Salivary Cortisol ELISA SLV-2930, DRG International, USA) with intra- and inter-assay coefficients of variation <6 and <9 %, respectively.

To correlate the DST with ICS dose, a standardized ICS dose of budesonide equivalent in μg/day was calculated from the dose and type of ICS the participant was using (100 μg beclomethasone extra fine = 200 μg beclomethasone = 100 μg fluticasone = 200 μg budesonide) [4]. Fractional suppression of cortisol after dexamethasone was calculated as ((pre-dex cortisol − post-dex cortisol)/pre-dex cortisol) × 100 (%).

A power calculation showed that, based on a 2-sided test, sample size 16, alpha 5 %, we had 80 % power to detect a 1 SD difference in post-dexamethasone salivary cortisol levels (~5 nmol/l) between test 1 and test 2.

Bland–Altman plots (the differences between two repeated measurements for each subject plotted against the mean of those measurements) were produced for pre- and post-dexamethasone cortisol levels. As a measure of repeatability, the 95 % limit of agreement was calculated. This indicates how far apart measurements on two different time points may be for individual patients. If differences within this limit are not clinically important, the measurement is considered reproducible.

## Results

23 Children (6 females), mean age 12.1 years (95 %CI 10.9–13.4), were included (Table 1). Most children (17/23) had lower salivary cortisol levels after dexamethasone, although 6/23 children showed an increase. Basal morning cortisol levels in test 1 and 2 correlated significantly (*r* = 0.66; *p* = 0.017).

**Table 1:** Clinical characteristics at baseline

	Total group	Reproducibility group
*N*	23	16
Age (years)	12.1 (10.9–13.4)	12.6 (11.2, 14.1)
Gender (m/f)	17/6	12/4
Height SDS	−0.5 (−1.1, 0.4)	−0.5 (−1.2, 0.2)
BMI SDS	0.6 (0.1, 1.1)	0.7 (0.0, 1.3)
ICS dose per kg (mcg)	11.0 (9.1, 12.9)	11.5 (9.2, 13.8)
Asthma control test score^a^	22 (21–24)	23 (21–24)
Compliance (ICS use in days per week)	6.5 (6.2–6.8)	6.4 (6.0–6.8)

All values expressed as mean (95 % CI), apart from gender

^a^A value of >19 is considered good asthma control

Cortisol levels after dexamethasone, change in cortisol levels and fractional suppression of cortisol after dexamethasone did not correlate between test 1 and 2 (*r* = 0.51; *p* = 0.1, *r* = 0.42; *p* = 0.1 and *r* = 0.28; *p* = 0.2, respectively). Basal salivary cortisol levels were below the reference (8–30 nmol/l) in 9/23 (39 %) and 8/16 (50 %) children at test 1 and 2, respectively. However, only 3 children had reduced levels in both tests.

**Fig. 1:**
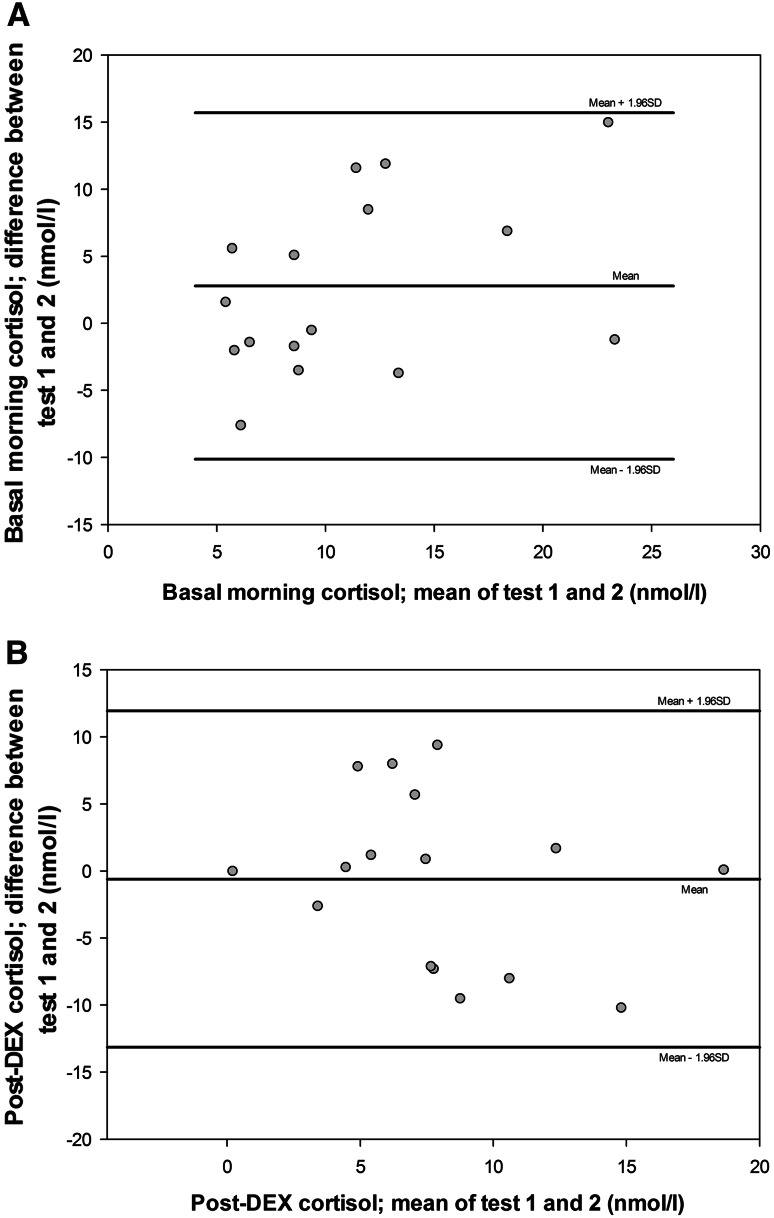
**a** Bland–Altman plot for salivary cortisol levels before dexamethasone test 1 vs. test 2. The *line* ‘mean’ indicates the mean difference between test 1 and 2 for all participants. Also, the 95 % CI for the difference between test 1 and 2 is indicated, showing that there is considerable variation between test 1 and 2, and thus poor reproducibility. **b** Bland–Altman plot for salivary cortisol levels after dexamethasone test 1 vs. test 2

Bland–Altman plots show that the mean difference for both basal and post-dexamethasone levels was not significantly different from zero, indicating no fixed bias between tests 1 and 2 (Fig. 1). The 95 % limits of agreement, indicating how far apart measurements on the two occasions were likely to be for most individuals, were 12.9 nmol/l for basal cortisol levels and 12.5 nmol/l for post-dexamethasone measurements.

ICS dose, uncorrected or expressed as μg/kg, μg/BSA or μg/age, did not correlate with baseline cortisol levels nor differed between those subjects with basal salivary cortisol levels below vs. within/above the reference. ICS dose and baseline cortisol levels did not correlate with height SDS or BMI SDS.

## Discussion

This pilot study is the first to investigate reproducibility and utility of the 0.25 mg DST using salivary cortisol as a marker for glucocorticoid sensitivity in children with asthma. We studied reproducibility by performing two low-dose DST’s in the same children, 1 month apart, without changing their maintenance ICS.

The low-dose or 0.25 mg DST is an innovative and easy applicable method to assess glucocorticoid sensitivity. Various authors reported associations with glucocorticoid sensitivity determining genotypes [5–8]. The test has been popular in psychology/psychiatry research, where some authors found correlations between the degree of cortisol suppression and clinical parameters [9, 10]. Both plasma [6, 8, 10] and salivary [5] cortisol measurements have been used in these studies. However, reproducibility of the low-dose DST has not received much attention in the scientific literature.

We found a good correlation, but low reproducibility of baseline cortisol levels. Our data indicate considerable variation in repeated measures of salivary cortisol, up to 12 nmol/l. As the analytical variation of the test is only 9 %, this suggests considerable within-subject (biological) variation. Common upper respiratory tract infections and/or psychosocial stress might have contributed to this variation. In addition, post-dexamethasone salivary cortisol levels, absolute change and fractional suppression of salivary cortisol levels after dexamethasone did not correlate between the two tests. This shows that the 0.25 mg DST had a poor reproducibility in our study.

Previous studies investigating the reliability of HPA-axis measures are scarce and most studies using low-dose DST’s have been performed in adults. Golden et al. reviewed reliability of several assessment methods of the HPA-axis [11]. In keeping with our findings, an 8 am salivary cortisol had the lowest between-visit reliability (*R* = 0.18–0.47). Reliability of a 0.5 mg DST was considered reasonable (*R* = 0.42–0.66). Wingenfeld et al. [12] also studied reproducibility of the 0.5 mg DST and found a significant correlation between the fractional cortisol suppression for a 4 pm sample, but only a borderline correlation for the 8 am sample. Because a higher dexamethasone dose will result in a greater or complete suppression of cortisol, the 0.5 mg DST is more likely to be reproducible. However, higher doses of dexamethasone will result in less variation in the degree of cortisol suppression and are therefore less suitable to determine glucocorticoid sensitivity. Reproducibility of the 0.25 mg DST, using either serum or saliva, was studied only once before, in adults, by Reynolds et al. [13]. In 29 healthy subjects, salivary cortisol levels (post-dexamethasone) had much more within-subject variation than plasma cortisol levels [13]. The authors suggested sampling problems (e.g. variable salivary flow rates) or a poor correlation between salivary and plasma cortisol levels at low concentrations (post-dexamethasone) as an explanation. Our study is the first to investigate reproducibility of the low-dose DST in children.

Reduced basal salivary cortisol levels have been reported in children and adults with asthma on steroid treatment [14, 15], suggesting that an effect of ICS on the HPA-axis can be measured with salivary cortisol samples. In our study, three children showed repeated basal cortisol levels below the reference. Lower basal cortisol levels in children with asthma could reflect partial adrenal insufficiency, which might warrant evaluation by paediatric endocrinology, if there are clinical symptoms.

In conclusion, our study shows that the low-dose salivary DST test in its current form is not suitable for use in clinical practice in children with asthma, due to low reproducibility. Therefore, studies using the 0.25 mg salivary DST should be interpreted cautiously.
